# Optimizing nursing care in phototherapy to improve treatment outcomes in neonatal jaundice management

**DOI:** 10.3389/fped.2025.1652188

**Published:** 2025-12-17

**Authors:** Qian Dong, Fan Huang

**Affiliations:** 1 Department of Neonatology, Wuhan Dongxihu District People’s Hospital, Wuhan, China; 2 Department of Pediatrics, Wuhan Dongxihu District People’s Hospital, Wuhan, China

**Keywords:** adverse events, bilirubin, neonatal jaundice, nursing care, phototherapy

## Abstract

**Objective:**

Neonatal jaundice is a common condition affecting many newborns and often requires phototherapy to manage elevated bilirubin levels. This study aimed to evaluate the impact of optimized nursing care during phototherapy on treatment outcomes, investigating common nursing challenges and assessing whether enhanced interventions could accelerate recovery and improve overall effectiveness.

**Methods:**

A total of 106 neonates with jaundice who underwent phototherapy between January 1 and December 1, 2023, were randomly assigned to a control group and an intervention group, with 53 cases in each. The control group received standard nursing care, while the intervention group received targeted nursing interventions addressing phototherapy-related issues. Relevant clinical and laboratory data were collected from hospital records and nursing assessments, and treatment outcomes were compared between the two groups.

**Results:**

The intervention group exhibited significantly shorter times for symptom resolution, complete blood count recovery, first defecation, and meconium transition to yellow compared to the control group (*P* < 0.05). Moreover, total and indirect bilirubin levels were significantly lower in the intervention group than in the control group (*P* < 0.05), while direct bilirubin showed no significant difference. The rate of adverse nursing events was significantly lower in the intervention group (1.89%) compared to the control group (13.21%) (*P* < 0.05). Additionally, the quality-of-care score for the intervention group was significantly higher than that of the control group (*P* < 0.05).

**Conclusions:**

Enhancing nursing interventions for phototherapy-related issues in neonatal jaundice significantly improves therapeutic outcomes, accelerates recovery, and optimizes the effectiveness of phototherapy.

## 1 Introduction

Neonatal jaundice is a common condition affecting approximately 60% of full-term and 80% of preterm newborns worldwide (1). The condition is multifactorial, with symptoms typically appearing between the second and eighth day of life. These symptoms are often attributed to immature liver function and impaired bilirubin metabolism, which can lead to serious complications such as bilirubin encephalopathy and kernicterus (2, 3). If not properly managed, neonatal jaundice can result in long-term neurological impairments, highlighting the importance of timely interventions to prevent complications and ensure healthy development (4). Phototherapy, a cornerstone treatment for neonatal jaundice, converts unconjugated bilirubin into water-soluble configurational isomers excreted without liver conjugation and structural isomers (lumirubin) excreted in bile and urine, thereby facilitating bilirubin elimination. However, phototherapy carries risks, including skin damage, dehydration, and potential adverse effects on the infant's physiological state (5, 6).

In recent years, optimizing nursing care for infants undergoing phototherapy has become increasingly important. Effective nursing interventions can help minimize adverse reactions, enhance treatment efficacy, and promote faster recovery (7). Effective nursing care during phototherapy involves close monitoring, individualized care plans, and early identification of potential risks to optimize neonatal safety and outcomes. Therefore, this study aimed to evaluate the effects of optimized nursing interventions on phototherapy outcomes in neonates with jaundice. Targeted nursing strategies were applied to neonates receiving phototherapy at our hospital. The study highlights the importance of evidence-based nursing practices in improving safety and treatment effectiveness and offers practical guidance for developing standardized care protocols for neonatal jaundice management.

## 2 Materials and methods

This study was designed as a randomized controlled trial to evaluate the effect of optimized nursing interventions on treatment outcomes in neonates undergoing phototherapy for jaundice. We hypothesized that targeted nursing care addressing phototherapy-related challenges would accelerate recovery, reduce adverse events, and improve overall treatment effectiveness compared to standard nursing care.

### 2.1 Participants

The study participants were selected from neonates diagnosed with jaundice and admitted to Wuhan Dongxihu District People's Hospital between January 1 and December 1, 2023. Eligible participants were recruited according to predefined inclusion and exclusion criteria and randomly assigned to the control or intervention group using a random number table. The diagnostic criteria for neonatal jaundice were based on the guidelines for neonatal jaundice in China (8), which include the following: (1) Jaundice occurring within 24 h of birth; (2) Total serum bilirubin levels reaching the phototherapy intervention threshold for the corresponding age and risk factors, or exceeding the 95th percentile of the bilirubin risk curve; bilirubin increasing by more than 85 µmol/L (5 mg/dL) daily or >0.5 mg/dL per hour; (3) Jaundice persisting for an extended period, with full-term infants having jaundice lasting longer than 2 weeks, and preterm infants longer than 4 weeks; (4) Jaundice recurs after subsiding; (5) Serum direct bilirubin ≥34 µmol/L (2 mg/dL), 20% of total serum bilirubin considered significant.

The inclusion criteria were: neonates diagnosed with jaundice requiring phototherapy, and parents or guardians who consented to participate in the study and signed the informed consent form. The exclusion criteria were: neonates who received additional interventions such as exchange transfusion or intravenous immunoglobulin, those with organ dysfunction or systemic infectious diseases, and those with incomplete clinical data.

### 2.2 Nursing care

The control group received routine care, where nurses followed departmental management protocols, providing medication guidance, health monitoring, and skin care. The intervention group received enhanced nursing interventions designed to address specific challenges in phototherapy care, which included the following:

#### 2.2.1 Analysis of common issues and underlying causes

Common adverse nursing events during phototherapy for neonatal jaundice include skin injuries, crying, and fever. The causes of these nursing issues are as follows:

1. Environmental changes: Exposure to light during phototherapy can reduce the infant's sense of security. Changes in the environment, along with light and temperature fluctuations, may result in crying, restlessness, and sweating.
2. Risk of skin damage: Newborn skin is delicate, and insufficient protective measures can increase the risk of injury. Scratching from untrimmed nails, friction from movement, and delayed diaper changes in a high-temperature environment can cause abrasions and diaper rashes. Extended phototherapy and friction from eye shields may exacerbate skin damage.
3. Body temperature fluctuations: Neonates, with immature thermoregulatory centers, are susceptible to overheating under phototherapy, resulting in symptoms such as flushing, crying, and agitation.
4. Fluid deficiency: Phototherapy accelerates water loss, and insufficient hydration can lead to dehydration.
5. Changes in feeding patterns: Separation from the mother during phototherapy may alter feeding patterns. Some infants may not adapt well to bottle-feeding, which can increase crying and irritability.
6. Lack of knowledge: Insufficient nurse training on phototherapy can result in improper equipment maintenance, failure to reposition infants to avoid single-sided light exposure, and decreased treatment effectiveness.

#### 2.2.2 Nursing quality improvement

Nursing quality improvement focused on the following aspects (6, 8):

1. Clinical Monitoring: Delayed meconium passage and inadequate milk intake can exacerbate jaundice. Nurses closely monitored bowel movements and feeding patterns during each shift, implementing interventions like enemas or anal dilation when necessary. Massage was also utilized as an adjunct to help alleviate jaundice.
2. Meeting Infant Needs: Environmental changes could cause discomfort, so nurses prioritized identifying the causes of distress, such as wet diapers, hunger, or an inappropriate incubator temperature. Nurses aimed to ensure the infant's safety and comfort, especially during phototherapy, when the infant was separated from parents. Gentle communication and physical contact were used to soothe the infant. For cases of intense crying or fatigue, mild sedatives were administered according to the physician's orders to help conserve energy and ensure smooth treatment.
3. Skin Care: Before phototherapy, nurses ensured the infant's skin was clean, nails were trimmed, and gloves and socks were provided to prevent restricted circulation. Diapers were changed promptly to avoid diaper rashes caused by the high temperature of the phototherapy environment. The perineal area was kept dry and clean, and the umbilical area was protected to prevent infection. The use of powders or oils, which could impair phototherapy efficacy, was avoided.
4. Regular Observation: Nurses regularly monitored the infants' skin for damage and checked infusion sites for leakage or dislodgement. Body temperature was measured frequently, and the phototherapy box was adjusted to prevent overheating or chilling. Ventilation was applied as needed, and therapy was paused if the temperature exceeded 38°C. Serum bilirubin levels were closely monitored, and any changes in jaundice were observed to assess the treatment's effectiveness. Nurses also monitored the infant's mental state and vital signs, paying particular attention to signs of bilirubin encephalopathy, such as changes in crying, sucking, or muscle tone. Any abnormalities, such as sweating, rashes, or changes in stool and urine, were addressed promptly.
5. Feeding and Hydration Care: Nurses carefully managed hydration and feeding to prevent dehydration, fever, and acidosis due to excessive sweating. Frequent small feedings were administered, and intravenous fluids were provided as needed according to medical orders. After feeding, infants were positioned in a side-lying posture to reduce the risk of aspiration or choking.
6. Blue Light Box Maintenance: The phototherapy equipment, including the blue light box, was regularly cleaned and disinfected. Distilled water was used in the humidifier tank, and air purification pads beneath the box were cleaned frequently. LED strips and reflective plates were inspected and cleaned to prevent dust buildup that could impair phototherapy efficacy. Detailed records of phototherapy duration and conditions were maintained, with treatment cessation when symptoms and signs returned to normal.

### 2.3 Observation indicators

Data for this study were collected through a combination of hospital electronic medical record review and structured nursing system assessments. Demographic information, laboratory test results, and other study-relevant data were extracted from the electronic medical records, while clinical observations and nursing evaluations were systematically recorded by trained nurses.

#### 2.3.1 Recovery progress

Recovery progress was assessed through various indicators, including time to symptom resolution, time for complete blood count recovery, including red blood cell count, hemoglobin level and reticulocyte count, time to first defecation, and time for meconium to turn yellow. These parameters were used to compare the speed and extent of recovery between the two groups.

#### 2.3.2 Bilirubin levels

Bilirubin levels were assessed by measuring total, direct, and indirect bilirubin. These parameters are crucial for evaluating the severity of jaundice and monitoring its resolution. Comparing these values between the two groups allowed us to assess the effectiveness of the treatment protocol.

#### 2.3.3 Adverse nursing events

Adverse nursing events, such as skin damage, excessive crying, and fever, were recorded and analyzed. These events are common in neonates undergoing phototherapy and can indicate potential issues with the care process. The occurrence of these events in both groups was compared to evaluate the safety and quality of care provided.

#### 2.3.4 Nursing quality

Nursing quality was evaluated based on adherence to established care protocols, health education provided to parents, and the overall execution of nursing tasks. The quality of care was quantified using a percentage-based scoring system, with higher scores reflecting better adherence to care standards and improved patient outcomes.

### 2.4 Statistical analysis

Statistical analysis was performed using SPSS version 22.0 (SPSS Inc., Chicago, IL, USA). The normality of the data was assessed using the Shapiro–Wilk test. For normally distributed continuous variables, data are presented as mean ± standard deviation, and intergroup comparisons were conducted using t-tests. For non-normally distributed continuous variables, data are presented as median (interquartile range), and non-parametric tests were used for intergroup comparisons. Categorical variables are presented as percentages (%), and *χ*² tests were used for group comparisons. A *P*-value of <0.05 was considered statistically significant.

## 3 Results

A total of 106 participants were included in the study and randomly assigned to two groups, with 53 infants in each group. In the control group, there were 30 male and 23 female infants, aged between 1 and 23 days, with a mean age of 5.24 ± 1.24 days. In the intervention group, there were 33 male and 20 female infants, aged between 1 and 18 days, with a mean age of 5.17 ± 1.36 days. Clinical characteristics between the two groups were comparable (*P* > 0.05). In addition, there were no significant differences in baseline bilirubin levels, including total bilirubin (275.20 ± 64.27 vs. 272.78 ± 60.74 μmol/L, *P* = 0.842), direct bilirubin (16.73 ± 7.89 vs. 15.86 ± 4.35 μmol/L, *P* = 0.484), and indirect bilirubin (253.85 ± 71.07 vs. 256.91 ± 59.29 μmol/L, *P* = 0.810).

### 3.1 Recovery progress

The recovery indicators, including time to symptom resolution, time for complete blood count recovery, time to first defecation, and time for meconium to turn yellow, were significantly shorter in the intervention group than in the control group (*P* < 0.05), as shown in Table 1.

**Table 1:** Comparison of recovery progress indicators between the two groups.

Group	Symptom resolution time	Blood count recovery time	First defecation time	Meconium yellowing time
Control Group (*n* = 53)	9.19 ± 1.48	7.33 ± 1.41	9.64 ± 1.29	54.19 ± 4.27
Intervention Group (*n* = 53)	7.23 ± 1.56	5.27 ± 1.33	6.48 ± 0.98	29.25 ± 2.16
*t*	8.312	9.344	11.289	16.345
*P*	0.005	0.003	0.001	<0.001

### 3.2 Bilirubin levels

After the intervention, total bilirubin (137.60 ± 38.56 vs. 163.67 ± 36.44 μmol/L) and indirect bilirubin (126.93 ± 42.64 vs. 154.15 ± 35.57 μmol/L) were significantly lower in the intervention group than in the control group (both *P* < 0.001), whereas direct bilirubin showed no significant difference (8.37 ± 4.73 vs. 9.52 ± 2.61 μmol/L, *P* = 0.124), as presented in Table 2.

**Table 2:** Comparison of bilirubin levels between the two groups.

Group	Total bilirubin	Direct bilirubin	Indirect bilirubin
	(μmol/L)	(μmol/L)	(μmol/L)
Control Group (*n* = 53)	163.67 ± 36.44	9.52 ± 2.61	154.15 ± 35.57
Intervention Group (*n* = 53)	137.60 ± 38.56	8.37 ± 4.73	126.93 ± 42.64
*t*	3.52	1.55	3.57
*P*	<0.001	0.124	<0.001

### 3.3 Rate of nursing adverse events

The incidence of nursing adverse events was significantly lower in the intervention group (1.89%) compared to the control group (13.21%) (*P* < 0.05), as shown in Table 3.

**Table 3:** Comparison of nursing adverse event rate between the two groups.

Group	Skin injury	Crying	Fever	Others	Total incidence rate
Control Group (*n* = 53)	1 (1.89%)	2 (3.77%)	1 (1.89%)	3 (5.66%)	13.21%
Intervention Group (*n* = 53)	0	0	0	1 (1.89%)	1.89%
*t*					4.867
*P*					0.027

### 3.4 Quality of nursing care

The quality of nursing care scores were significantly higher in the intervention group than in the control group, with notable differences between the two groups (*P* < 0.05), as shown in Table 4.

**Table 4:** Comparison of nursing quality scores between the two groups.

Indicator	Control Group	Intervention Group	*t*	*P*
Nursing safety	87.36 ± 1.21	93.26 ± 2.78	13.348	<0.001
Technical skills	86.59 ± 1.26	92.59 ± 3.15	14.716	<0.001
Work standards	86.41 ± 1.12	95.12 ± 3.15	14.448	<0.001
Protocol adherence	87.59 ± 1.36	96.45 ± 1.29	15.269	<0.001
Service attitude	87.87 ± 1.65	95.33 ± 2.26	15.379	<0.001
Health education	86.21 ± 1.46	96.48 ± 2.37	13.165	<0.001

## 4 Discussion

Neonatal jaundice is a common condition in newborns, characterized by the yellowing of the skin and sclera due to elevated bilirubin levels in the body, which is primarily caused by an increase in unconjugated bilirubin. Phototherapy is a well-established adjunctive treatment for neonatal hyperbilirubinemia (1, 5, 9, 10). It uses fluorescent light to convert unconjugated bilirubin into water-soluble isomers that are then excreted through bile and urine. When combined with appropriate nursing interventions, phototherapy can significantly enhance overall treatment outcomes.

In this study, the recovery progress indicators in the intervention group were notably better than those in the control group, suggesting that specific interventions in neonatal jaundice phototherapy nursing can accelerate recovery. Following the principle of “three parts treatment, seven parts nursing,” comprehensive nursing care, including appropriate feeding (breastfeeding or formula feeding) and skin care, works synergistically to optimize treatment outcomes (11). Addressing neonatal comfort and safety can reduce anxiety, minimize complications, and shorten hospital stays, thereby improving resource utilization. Nurses play a critical role in developing individualized phototherapy care plans tailored to the specific needs of neonatal jaundice patients. They carefully manage environmental factors, feeding routines, and other critical aspects while emphasizing personalized and humanistic care. Effective communication with physicians is also essential to ensure that any changes in the newborn's condition are promptly addressed. Throughout the phototherapy process, nurses must regularly adjust care strategies to ensure adequate hydration and nutrition. This includes offering small, frequent feedings and providing water during feeding intervals, especially glucose water. In hot weather, it is particularly important to prevent dehydration and heat-related issues, which may require intravenous fluid therapy for infants unable to feed orally. Nurses must also maintain the proper temperature and air quality in the phototherapy units, meticulously document treatment details, and adhere to specific guidelines for discontinuing phototherapy. This systematic approach ensures optimal care management, streamlined nursing workflows, and improved comfort and health outcomes for newborns undergoing phototherapy for jaundice (12).

The bilirubin levels in the intervention group was improved compared to the control group, indicating that the nursing interventions had a positive effect on the prognosis of neonatal jaundice. Nurses must strengthen their sense of responsibility by continually monitoring changes in the newborn's condition and vital signs. By adjusting treatment and care plans as needed, nurses can address both the physiological and psychological needs of jaundiced infants. Nurses are encouraged to stay updated on specialized knowledge and skills to provide effective clinical care (13, 14). Nurses play a key role in evaluating phototherapy efficacy by closely observing the distribution, severity, and dynamic changes of jaundice. Blood bilirubin should be measured with the phototherapy lights turned off to avoid inaccurate readings, and additional tests should be performed within four hours after stopping phototherapy to detect any rebound hyperbilirubinemia. These monitoring measures allow timely adjustment of medical interventions and help ensure safe and effective management of neonatal jaundice.

The lower incidence of adverse nursing events in the intervention group suggests that the implemented interventions enhanced the safety and reliability of phototherapy. Nurses should adopt a problem-specific approach to analyze the causes of adverse events and take targeted nursing interventions to enhance the phototherapy care management system. The intervention model for phototherapy care in neonatal jaundice should integrate modern service philosophies, such as patient-centered care and evidence-based practices, to continuously improve the care process. By identifying and addressing risk factors for poor nursing practices, nurses can reduce errors, alleviate physiological and psychological stress on infants, and reduce the adverse effects associated with phototherapy (15, 16).

The quality of nursing care in the intervention group was also significantly higher than in the control group, reflecting the positive impact of the intervention on the overall quality of care. Nurses should not only provide high-quality, comprehensive care during phototherapy but also address common nursing issues to enhance treatment safety and effectiveness. Educating and managing the families of the infants is crucial, as it improves their understanding of the healthcare process and fosters active participation in the care plan (17–19). By improving the care model, adhering to strict nursing protocols, and offering meticulous, human-centered care, nurses can create a supportive environment for both infants and their families. Through enthusiasm and skillful practice, nurses can build trust with families, address their concerns, provide clear explanations, and ensure that families are well-informed, all of which help reduce anxiety.

This study has several strengths, including its randomized controlled design, systematic data collection from hospital records and structured nursing assessments, and comprehensive evaluation of recovery, bilirubin levels, adverse events, and nursing quality. These aspects provide robust evidence for the effectiveness of optimized nursing interventions during phototherapy. However, the study also has limitations. It was conducted at a single center with a relatively small sample size, which may limit the generalizability of the findings. Future multicenter studies with larger cohorts are recommended to validate these results and further refine nursing protocols to enhance neonatal care outcomes.

## 5 Conclusion

In conclusion, strengthening nursing interventions in neonatal jaundice phototherapy can significantly improve treatment outcomes, accelerate recovery, and enhance the overall health of affected infants. By integrating individualized care plans, effective communication, and continuous monitoring, this approach not only reduces complications and shortens hospital stays but also optimizes the utilization of medical resources. The model for improving phototherapy care provides valuable insights for advancing neonatal jaundice management and should be explored further to ensure continuous improvements, ultimately contributing to better long-term health outcomes for affected infants.

## Data Availability

The raw data supporting the conclusions of this article will be made available by the authors, without undue reservation.
